# Design of Peptide-Modified Aluminum Nanoparticles with Enhanced Antimicrobial, Antibiofilm, Antioxidant, and DNA-Cleaving Properties

**DOI:** 10.3390/pharmaceutics17111490

**Published:** 2025-11-19

**Authors:** Zeynep Kanlidere, Nazlı Farajzadeh Öztürk, M. Serkan Yalçın, Sadin Özdemir

**Affiliations:** 1 Department of Pharmaceutical Basic Sciences, Faculty of Pharmacy, Acibadem Mehmet Ali Aydinlar University, 34752 Istanbul, Türkiye; 2 Department of Analytical Chemistry, Faculty of Pharmacy, Acibadem Mehmet Ali Aydinlar University, 34752 Istanbul, Türkiye; nazli.ozturk@acibadem.edu.tr; 3 Department of Chemistry and Chemical Processing Technologies, Technical Science Vocational School, Mersin University, 33343 Mersin, Türkiye; serkanyalcin@mersin.edu.tr; 4 Food Processing Programme, Technical Science Vocational School, Mersin University, 33343 Mersin, Türkiye; sadinozdemir@mersin.edu.tr

**Keywords:** antimicrobial, aluminum oxide nanoparticles, synthetic peptides, antioxidant, antidiabetic

## Abstract

**Background/Objectives**: In this study, three antimicrobial peptides (**1**–**3**) were conjugated onto bare aluminum nanoparticles (**NP**) to produce peptide-conjugated nanoparticles (**NP1**–**NP3**) in order to evaluate their biological effects. **Methods**: The peptide-functionalized Al_2_O_3_ nanoparticles were characterized and subsequently analyzed for their antimicrobial activity against selected bacterial strains. The findings were compared with those of bare Al_2_O_3_ nanoparticles and free antimicrobial peptides. Through this comparison, the enhanced impact of combining nanoparticles with peptides in addressing antimicrobial resistance was demonstrated. Additionally, biofilm inhibition, microbial cell viability inhibition, DNA cleavage, antioxidant, and amylolytic activity assays were performed to comprehensively evaluate the biological functionality of the synthesized nanoparticles. **Results**: Although all tested samples exhibited significant antimicrobial activity, peptide-conjugated nanoparticles **NP1**, **NP2**, and **NP3** provided superior activity with an MIC value of 16 mg/L. The highest biofilm inhibition activities were observed for **NP2** as 53% and 70% against *S. aureus* and *P. aeruginosa*, respectively. Additionally, **NP1**–**NP3** inhibited microbial cell viability by 100% at a concentration of 6.25 mg/L and free peptide **3** displayed *E. coli* inhibition as 100% at a concentration of 12.5 mg/L. Furthermore, we evaluated the biological potential of antimicrobial peptide-functionalized Al_2_O_3_ nanoparticles through antibiofilm, antioxidant, antidiabetic activities, and DNA cleavage assays. Peptide-conjugated nanoparticles **NP1**, **NP2**, and **NP3** exhibited the highest antioxidant activities as 43.70%, 45.22%, and 59.57%, respectively. Except for **NP3**, the compounds were observed to act as α-amylase enzyme activators. **NP** and **NP1**–**NP3** completely degraded the supercoiled circular form into small pieces. **Conclusions**: Our findings suggest that peptide–aluminum nanoparticle conjugation may be a promising formulation for enhancing biological activity. Further in vitro and in vivo tests may help clarify the therapeutic potential of this novel nanoformulation.

## 1. Introduction

Infectious diseases are a big challenge for scientists and remain a major public health concern [1]. Antibiotics play an important role in helping the immune system fight harmful bacteria. They have become a key part of modern healthcare and have greatly reduced illness caused by infections [2]. However, overuse and misuse of antibiotics have led to the emergence and spread of treatment-resistant bacterial strains, which can result in fatalities and incurable illnesses [3]. As a result, scientists employ a variety of strategies to discover new antimicrobial treatments, such as using natural materials, developing unique synthetic compounds, and repurposing current drugs to address resistance issues.

In recent years, antimicrobial peptides (AMPs) have gained attention as potential new treatments for infectious diseases [4,5]. The primary mechanism of action of AMPs is causing structural disruption in the cell membrane [6]. One of the key features of AMPs is their amphipathic structure, which means they have both hydrophilic and hydrophobic parts. This allows them to interact easily with cell membranes. Because AMPs are usually positively charged, they are strongly attracted to the negatively charged membranes of bacteria. This electrostatic attraction helps them bind to and damage bacterial cells while leaving human cells mostly unharmed. By directly affecting the structure and function of microbial membranes, AMPs make it difficult for pathogens to survive or develop resistance. This makes them promising candidates for developing new types of antimicrobial drugs in the future. 

Despite their strong antimicrobial potential, peptides have several drawbacks. These include susceptibility to enzymatic degradation, limited stability under physiological conditions, and potential cytotoxicity at high concentrations. Therefore, enhancing their stability and bioavailability is a key focus in the development of effective antimicrobial therapies. In recent years, nanotechnology has offered innovative solutions to overcome these limitations [7]. Among metal nanoparticles, AMPs conjugated with silver (Ag) and gold (Au) have been extensively studied due to their strong antimicrobial properties [8,9]. Compared to these, there are only a limited number of studies in the literature on AMPs conjugated with aluminum. Both antimicrobial peptides and aluminum oxide, have individually demonstrated activity against resistant bacterial strains [10,11,12]. However, studies combining synthetic peptides with Al_2_O_3_ nanoparticles for this purpose remain scarce.

Aluminum oxide (Al_2_O_3_) nanoparticles, are well-known for their broad-spectrum antimicrobial activity [13]. In addition, their high surface area, chemical and thermal stability, mechanical strength, biocompatibility, and ability to generate reactive oxygen species contribute to their antimicrobial activity. These properties enable their use in diverse sectors, including implantable medical devices, advanced water purification technologies, protective food-contact materials, and antimicrobial surface treatments [14]. However, similar to AMPs, Al_2_O_3_ nanoparticles also face challenges such as colloid stability and cytotoxicity [15].

To address these limitations, the conjugation of AMPs to aluminum oxide nanoparticle surfaces has emerged as a promising strategy. A few studies have investigated the conjugation of antimicrobial peptides onto aluminum oxide surfaces to enhance their antibacterial properties [16,17]. Additionally, one study has reported the use of peptide-functionalized aluminum oxide in the development of a biosensor for endotoxin detection [18]. 

In our study, we synthesized antimicrobial peptides and functionalized the surface of Al_2_O_3_ nanoparticles with these peptides to develop a hybrid nanomaterial with antimicrobial performance. In addition to the antimicrobial effect, we investigated the broader biological potential of AMP-functionalized Al_2_O_3_ nanoparticles including antibiofilm, antioxidant, antidiabetic, and DNA cleavage activities. To the best of our knowledge, this is the first report of peptide-modified aluminum oxide nanoparticles being evaluated for such a comprehensive range of biological activities, demonstrating significant promise for biotechnological applications.

## 2. Materials and Methods

### 2.1. Materials

#### 2.1.1. Synthesis and Characterization

Unless otherwise specified, all chemicals and reagents used were of analytical grade. For peptide synthesis, Fmoc-protected L-amino acids, coupling reagents, and Rink amide resin were purchased from CEM Corporation (Charlotte, NC, USA). HPLC-grade acetonitrile and methanol were purchased from Merck (Darmstadt, Germany), while DMF, DIC, Oxyma^®^, piperidine, DCM, and TFA were obtained from Sigma-Aldrich (Darmstadt, Germany). Aluminum nitrate Al(NO_3_)_3_·9H_2_O and sodium hydroxide (NaOH) pellets (pure analytical reagents) were also purchased from Merck (Darmstadt, Germany).

#### 2.1.2. In Vitro Experiments

2,2-Difenil-1-pikrilhidrazil (DPPH), *pBR322* plasmid DNA, 0.5× TBE (Tris-Borat-EDTA), Nutrient Broth (NB), NB agar, agarose, and crystal violet were obtained from Sigma-Aldrich.

#### 2.1.3. Bacterial Strains and Cells

*Staphylococcus aureus* (ATCC 6538), *Bacillus spizizenii* (ATCC 6633), *Enterococcus faecalis* (ATCC 29212), *Escherichia coli* (ATCC 10536), *Pseudomonas aeruginosa* (ATCC 27853), *Legionella pneumophila* subsp. pneumophila (ATCC 33152), *Candida albicans* (ATCC 15126), and *Candida glabrata* (ATCC 15126) were used as bacterial and fungal strains.

### 2.2. Methods

#### 2.2.1. Peptide Synthesis and Characterization

Peptides **1**–**3** were synthesized using a microwave-assisted automated peptide synthesizer (CEM Liberty system), following standard Fmoc-based solid-phase peptide synthesis (SPPS) protocols. The synthesis was carried out in accordance with the protocol provided by CEM Corporation. Rink amide resin (loading capacity: 0.7 mmol/g loading, as specified by the supplier) was used at 0.1 mmol scale for all syntheses. Amino acid coupling was performed by adding Fmoc-protected amino acids (0.25 mmol, 0.2 M in DMF), OxymaPure (0.25 mmol, 0.5 M in DMF), and DIC (0.50 mmol, 0.5 M in DMF) followed by microwave heating at 90 °C for 2 min (single coupling) or 2 × 2 min (double coupling). N-terminal Fmoc deprotection was carried out using 20% (*v*/*v*) piperidine in DMF at 90 °C for 1.5 min.

After synthesis, the cleavage was carried out for 30 min at 38 °C using a cleavage cocktail composed of TFA/TIS/H_2_O in a ratio of 95:2.5:2.5. After cleavage, the reaction mixture was slowly added into cold ethyl ether to precipitate the peptide. The resulting suspension was centrifuged, producing the crude peptide as a white solid. All peptides were lyophilized overnight. The peptide solutions were purified by reversed-phase high performance liquid chromatography (RP-HPLC, 1260 Infinity, Agilent Technologies Inc., Santa Clara, CA, USA). Analytical separations were performed at a flow rate of 1 mL/min using a gradient elution of 5 to 80% acetonitrile. The mobile phase consisted of solvent A (0.05% trifluoroacetic acid in water) and solvent B (0.05% trifluoroacetic acid in acetonitrile). Peptides were analyzed by liquid chromatography-mass spectrometry (LC-MS)/MS system (6420 Triple Quad, Agilent Technologies, Inc., Santa Clara, CA, USA).

#### 2.2.2. Preparation of Aluminum Nanoparticles

Aluminum oxide nanoparticles were synthesized as described in the literature with some modifications [19,20]. Briefly, a 100 mL aqueous solution of Al(NO_3_)_3_·9H_2_O (0.4 M) was heated to 80 °C. Then, 50 mL of NaOH (3 M) was added dropwise to the vigorously stirred Al(NO_3_)_3_ solution. The obtained aluminum hydroxide was centrifuged and dried in an oven at 80 °C for 24 h. Aluminum nanoparticles were obtained after calcination of the white powder (aluminum hydroxide) at 450 °C for 8 h. The resultant nanoparticles were cooled to room temperature and kept in a desiccator.

#### 2.2.3. Surface Modification of Aluminum Nanoparticles with Peptides

Aluminum nanoparticles (10 mg) were dissolved in 9 mL distilled water and heated to 60 °C. A total of 1 mL of each aqueous solution of cationic peptide (**1**–**3**) was added to the solution of aluminum nanoparticles dropwise, stirred for 3 h, and collected after centrifugation.

#### 2.2.4. Physicochemical Characterization of Nanoparticles

The prepared nanoconjugates (**NP1**–**NP3**) were characterized using Fourier-transform infrared spectroscopy, scanning electron microscopy, transmission electron microscopy, and zeta potential analysis.

##### Fourier Transform Infrared Spectroscopy (FTIR)

FTIR spectra of the samples were recorded using a Thermo Scientific Nicolet iS10 spectrometer (Thermo Fisher Scientific, Waltham, MA, USA) to identify the characteristic functional groups and confirm possible interactions between peptides and nanoparticles. The samples were analyzed over the spectral range of 4000–400 cm^−1^ with a spectral resolution of 4 cm^−1^, averaging 32 scans per measurement to improve the signal-to-noise ratio. All spectra were baseline-corrected and processed using the instrument’s OMNIC software.

##### Scanning Electron Microscopy (SEM)

The surface morphology of the nanoparticles was examined using a Thermo Scientific Quattro scanning electron microscope (Thermo Fisher Scientific, Waltham, MA, USA). SEM images were acquired at an accelerating voltage of 5–20 kV and magnifications ranging from 10,000× to 100,000× under high-vacuum mode.

##### Energy Dispersive X-Ray Spectroscopy (EDS)

Elemental composition and distribution of the nanoparticles were analyzed using Energy-Dispersive X-ray Spectroscopy (EDS) attached to the Thermo Scientific Quattro scanning electron microscope (Thermo Fisher Scientific, Waltham, MA, USA) equipped with an EDAX EDS analyzer (AMETEK Inc., Berwyn, PA, USA). The analyses were carried out at an accelerating voltage of 15–20 kV under high-vacuum mode to ensure optimal X-ray generation and detection. Spectra were collected from several regions of each sample to ensure representative elemental composition. Data acquisition and quantification were carried out using the EDAX Genesis software (AMETEK Inc., Berwyn, PA, USA).

##### Transmission Electron Microscopy (TEM)

The morphology of the nanoparticles was examined using a Thermo Scientific Talos L120C transmission electron microscope (Thermo Fisher Scientific, Waltham, MA, USA) operated at an accelerating voltage of 120 kV. For sample preparation, a dilute dispersion of nanoparticles in ethanol was ultrasonicated for a few minutes to ensure uniform distribution, and a drop of the suspension was placed onto a carbon-coated copper grid (200 mesh). The grid was air-dried at room temperature before imaging. 

##### Zeta-Potential

Particle size measurements were performed using a Litesizer 500 instrument (Anton Paar, Graz, Austria) at room temperature). For zeta potential analysis, nanoparticle samples were appropriately diluted with deionized water. Measurements were carried out at 25 °C. Measurements were performed in disposable polystyrene cuvettes for size determination and folded capillary cells for zeta potential analysis. Each measurement was repeated three times, and the average values with standard deviations were reported.

#### 2.2.5. Antimicrobial Activity

The antibacterial properties of bare aluminum nanoparticle (**NP**), free peptides (**1**–**3**), and nanoconjugates (**NP1**–**NP3**) were assessed using the microdilution method. *Staphylococcus aureus*, *Bacillus spizizenii*, *Enterococcus faecalis*, *Escherichia coli*, *Pseudomonas aeruginosa*, *Legionella pneumophila* subsp. *pneumophila*, *Candida albicans*, and *Candida glabrata* were the microorganisms employed to assess antibacterial activity. One day before the experiments, the cultures were made from scratch. This process was applied to activate the microbiological cultures. Then, the microbial cultures were diluted with Nutrient Broth (NB) to a concentration range of 10^8^–10^9^ CFU/mL. A total of 150 µL of NB medium was added to all microplate wells. The first well contained 1024 mg/L of the tested sample and was serially diluted with a 1:1 ratio. Microbial inoculation was carried out after dilution. Microplates were incubated at 37 °C for 24 h. Then, minimum inhibition concentration (MIC) values were determined. The lowest concentration at which the compounds prevented microbial growth is defined as the MIC value.

#### 2.2.6. Biofilm Inhibition Activity

To test the ability of bare aluminum nanoparticle (**NP**), free peptides (**1**–**3**), and nanoconjugates (**NP1**–**NP3**) to inhibit biofilm formation by *S. aureus* and *P. aeruginosa* at concentrations of 5, 10, and 15 mg/L, 24-well plates were used. Fresh suspensions of each bacterial species were inoculated into 24-well plates containing Nutrient Broth (NB) medium after **NP**, **1**–**3**, and **NP1**–**NP3** compounds were added separately. To enable cell attachment to the surface, they were subsequently incubated for 72 h at 37.5 °C. Following incubation, 200 µL of phosphate-buffered saline (PBS) was used to thoroughly wash the biofilm-coated wells of the 24-well plate twice. For half an hour, they were left to air dry. Following these treatments, 200 μL of 1% aqueous crystal violet (CV) solution was applied to each plate well to assess the production of biofilms. For an hour, the biofilm was left to discolor. The CV was then removed from the plates by washing them with PBS. To recover the absorbed CV, ethanol was then added, and the plates were allowed to sit at room temperature for fifteen minutes. Using the following Equation (1), biofilm inhibition was measured at 595 nm using a spectrophotometer.

Biofilm Inhibition (%) = (A_control_ − A_sample_/A_control_) × 100 (1)

#### 2.2.7. Microbial Cell Viability Inhibition

Microbial cell viability inhibition by **NP**, **1**–**3**, and **NP1**–**NP3** was assessed using a slightly modified version of the method described by Ozay et al. [21]. The ability of **NP**, **1**–**3**, and **NP1**–**NP3** to suppress microbial cell viability was examined using *E. coli* strain (ATCC 25922). Initially, *E. coli* was inoculated into Nutrient Broth (NB) and incubated at 37.5 °C with shaking at 150 rpm for 24 h. The culture was then washed twice with sterile distilled water and centrifuged. Subsequently, *E. coli* cells were exposed to various concentrations of **NP**, **1**–**3**, and **NP1**–**NP3** for 90 min at 37.5 °C. After incubation, the treated samples were serially diluted and plated on Nutrient Agar. Plates were incubated at 37.5 °C for 24 h. Following incubation, bacterial colonies were counted, and microbial cell viability was calculated using Equation (2).

Cell viability inhibition (%) = (A_control_ − A_sample_/A_control_) × 10 (2)

#### 2.2.8. DNA Cleavage Activity

The supercoiled DNA cleavage activities of **NP**, **1**–**3**, and **NP1**–**NP3** samples were investigated by performing an agarose gel electrophoresis assay. Super-coiled *pBR322* plasmid DNA was used as a target DNA molecule. Various concentrations of **NP**, **1**–**3**, and **NP1**–**NP3** samples ranging from 32 mg/L to 128 mg/L were prepared. A total of 15 µL of **NP**, **1**–**3**, and **NP1**–**NP3** samples and 5 µL of pBR322 plasmid DNA were mixed in PCR tubes and incubated for 2 h at 37 °C in a dark place. The gel was prepared with 1% agarose and placed in an electrophoresis tank. Tris-acetate-EDTA buffer was added to the tank until the gel reached the top of the tank. After the addition of loading dye (3 µL) to the samples of **NP**, **1**–**3**, and **NP1**–**NP3**, the samples were loaded into the gel wells. The (+) and (−) ends of the electrophoresis tank were connected. Electrophoresis was run at 100 V for 1 h. The gel was then carefully removed from the tank, and a UV transilluminator was used to visualize the gel.

#### 2.2.9. Antioxidant Activity

By contrasting it with the antioxidant activity of ascorbic acid and trolox as standard, the free radical scavenging capacity of **NP**, **1**–**3**, and **NP1**–**NP3** was determined. The tubes were filled with 2,2-diphenyl-1-picrylhydrazyl (DDPH) solution in methanol (1 mL, 0.1 mM). They were incubated for 30 min at room temperature in a dark atmosphere after 0.25 mL of the appropriate doses of **NP**, **1**–**3**, and **NP1**–**NP3** were applied. Trolox and ascorbic acid served as positive controls. After 30 min, the absorbances measured at a wavelength of 517 nm were used to compute the scavenging ability using the following Equation (3).

DPPH radical scavenging ability % = [(Abs_control_ − Abs_sample_)/Abs_control_] × 100 (3)

#### 2.2.10. Amylolytic Activity

**NP**, **1**–**3**, and **NP1**–**NP3** were tested at concentrations of 6.25, 12.5, 25, 50, and 100 mg/L in a test tube. Phosphate buffer and α-amylase were added, and the mixture was incubated at 37 °C for 15 min. Afterwards, 0.2 mL of 1% potato starch solution was added to start hydrolysis. After another 20 min incubation at 37 °C, 0.4 mL of 3.5% dinitrosalicylic acid (DNS) was added to stop the hydrolysis. The tubes were then boiled in water for 5 min. A control without test samples was prepared. Following cooling, the mixture was diluted with 3 mL of distilled water, and absorbance was measured at 540 nm. The amylolytic activity was calculated using the following Equation (4).

Amylolytic Activity (%) = 100 − (Control_abs_ − Sample_abs_)/Control_abs_ × 100 (4)

## 3. Results and Discussion

### 3.1. Synthesis and Characterization of Peptides

The design of peptide sequences was made with consideration of the necessity of a cationic structure, which plays a significant role in the effectiveness of antimicrobial peptides (AMPs). Many AMP sequences are rich in positively charged amino acids with basic side chains, such as lysine and arginine [22]. These amino acids interact electrostatically with the negatively charged surfaces of microbial cell membranes, leading to membrane disruption and subsequent cell death [23]. Therefore, the inclusion of such amino acids in the peptide sequence was preferred to enhance antimicrobial activity.

In addition to the positive charge, the amphipathic nature of many AMPs was also taken into consideration in the design of the peptide sequence. Amphipathicity is defined by the presence of both hydrophilic and hydrophobic regions within the peptide structure. This dual characteristic allows the peptide to maintain solubility in aqueous environments while interacting with microbial membranes and facilitating membrane insertion.

Moreover, the length of peptide sequence was also considered during the design process. The sequence was kept as short as possible to minimize synthesis cost. Amino acid sequences consisting of ten or more residues are generally considered a safe threshold for maintaining antimicrobial activity in AMPs. For this reason, the peptide was designed to include at least ten amino acids. At the same time, the sequence was kept as short as possible to minimize synthesis cost.

**Figure 1:**
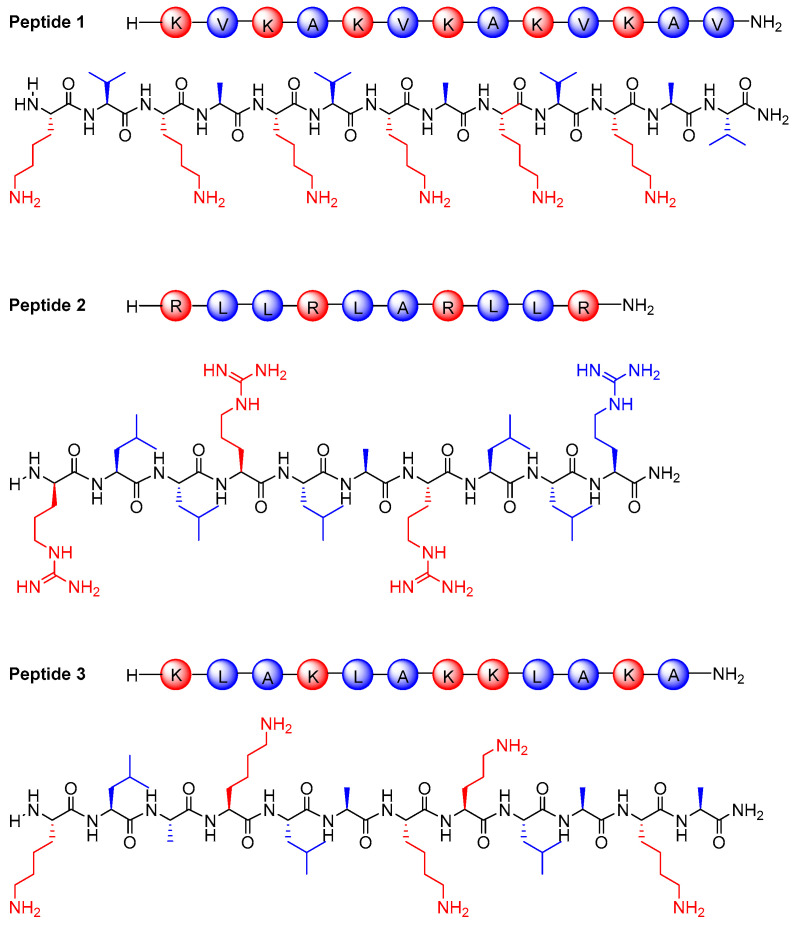
Chemical structures of peptides **1**–**3** studied in this work. Hydrophobic amino acids (leucine, valine, and alanine) are shown in blue, while positively charged amino acids (arginine and lysine) are shown in red.

For these reasons, peptides **1**–**3** were designed and synthesized using hydrophobic leucine (L), valine (V), or alanine (A) amino acids and positively charged arginine (R), or lysine (K) amino acids that target the bacterial cell membrane. The following peptides were synthesized (Figure 1): **1** (KVKAKVKAKVKAV-NH_2_), **2** (RLLRLARLLR-NH_2_), **3** (KLAKLAKKLAK-NH_2_).

Following synthesis, the peptides were first analyzed by LC-MS/MS to verify their molecular weights. The detection of molecular ion peaks with +2 and +3 charge states in the MS spectra validated the proper synthesis of peptides **1**–**3**. Subsequently, the corresponding peaks were collected by high-performance liquid chromatography (HPLC) based on their molecular weights. The calculated monoisotopic *m*/*z* values for the fragment ions of peptides **1**–**3** are listed in Table 1, and the MS spectra of the peptides are provided in the supplementary information (Figures S1–S3).

### 3.2. Preparation and Characterization of Peptide-Conjugated Aluminum Oxide Nanoparticles

As peptides are subject to proteolytic degradation in the bloodstream as well as the gastrointestinal tract, the development and utility of peptide-based systems as therapeutic agents have been limited. However, conjugation of peptides with nanoparticles is considered an efficient strategy to increase their stability and to extend the systemic half-life [24]. Therefore, the surface of aluminum oxide nanoparticles (NP) was modified with the newly synthesized peptides (**1**–**3**) in this study.

Following the successful synthesis and characterization of the peptides, aluminum oxide nanoparticles (NP) were synthesized and subjected to surface modification using the resultant peptides via non-bonding interactions. (See Methods, Section 2.2.3 and Section 2.2.4). The morphological and structural features of both unmodified (**NP**) and modified aluminum oxide nanoparticles (**NP1**–**3**) were examined using various microscopic, spectroscopic, and zeta potential measurements [25,26,27].

**Figure 2:**
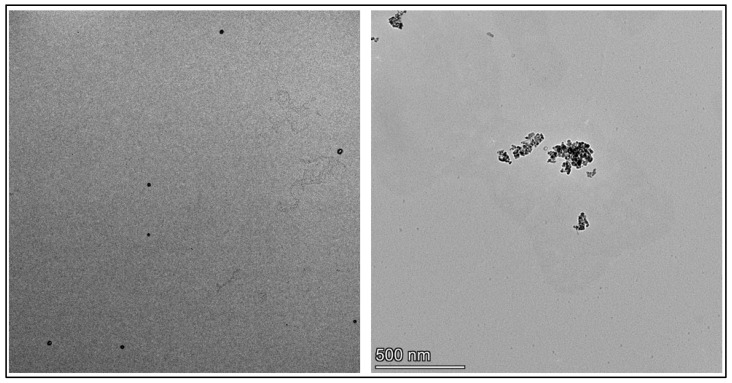
The TEM images of unmodified aluminum oxide nanoparticles (**NP**) (**left column**) and peptide modified aluminum oxide nanoparticles (**NP1**) (**right column**).

As a primary technique, transmission electron microscopy (TEM) was utilized to assess the size distribution, morphological features, and aggregation patterns of **NP** and **NP1**–**NP3**. TEM analysis revealed that the unmodified nanoparticles (**NP**) had a nearly spherical morphology with an average diameter of approximately 20–25 nm. TEM analysis further revealed that conjugation of aluminum oxide nanoparticles with peptides **1**–**3** induced noticeable aggregation compared to the unmodified nanoparticles. The observed aggregation may result from intermolecular hydrogen bonding and electrostatic interactions among peptide-coated nanoparticles, a typical behavior of peptide-functionalized nanostructures. TEM images of the unmodified nanoparticle (**NP**) and the peptide-functionalized nanoconjugate **NP1** are presented in Figure 2, providing visual evidence of the surface modification.

**Figure 3:**
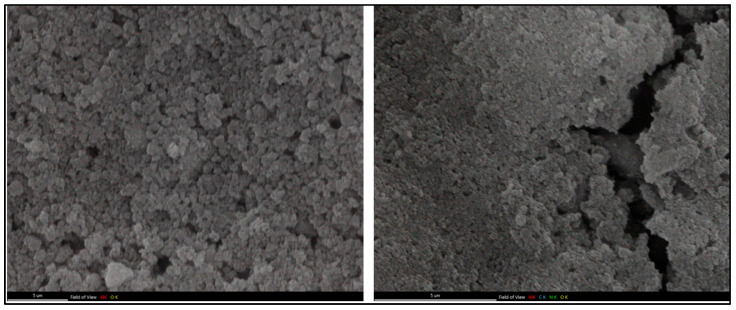
FESEM images of unmodified aluminum oxide nanoparticles (**NP**) (**left column**) and peptide-modified aluminum oxide nanoparticles (**NP1**) (**right column**).

TEM analysis was followed by further investigation of the morphologies of unmodified and peptide-modified aluminum oxide nanoparticles using field emission scanning electron microscopy (FESEM) and energy-dispersive spectroscopy (EDS). The FESEM data obtained for the unmodified aluminum oxide nanoparticle (**NP**) and the nanoconjugate **NP1** are provided in Figure 3. A significant morphological change observed on the surface of aluminum oxide nanoparticles confirmed the successful coverage of the surface of aluminum oxide with peptide **1** through non-covalent interactions. The textured surface of the bare nanoparticles became smoother after peptide attachment, suggesting the formation of a covarage on the Al_2_O_3_ surface.

**Figure 4:**
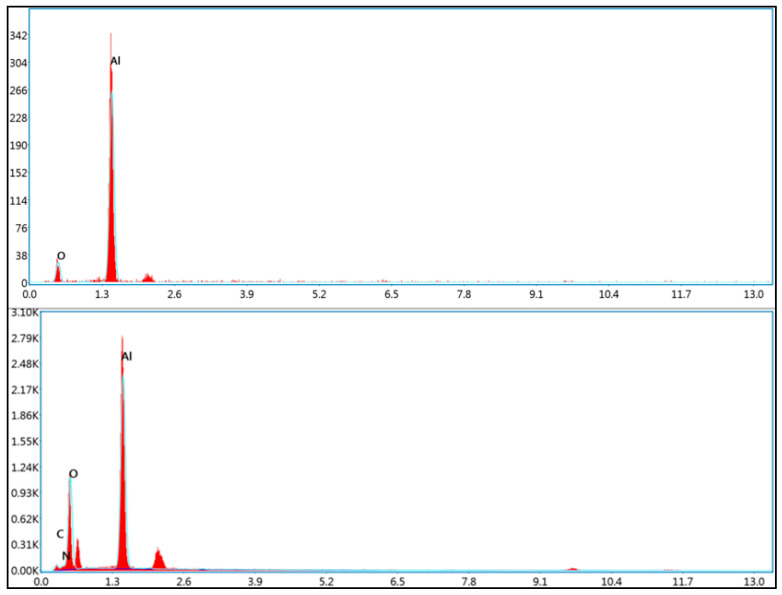
The elemental mapping analysis of unmodified aluminum oxide nanoparticles (**NP**) (**top**) and peptide-modified aluminum oxide nanoparticles (**NP1**) (**bottom**).

Furthermore, The EDS elemental mapping confirmed the presence and uniform distribution of oxygen, carbon, nitrogen, and aluminum on the surface of the peptide-modified nanoparticles. The detection of carbon and nitrogen, which are not present in the bare aluminum oxide structure, supports the successful conjugation of the peptide layer (Figure 4).

**Figure 5:**
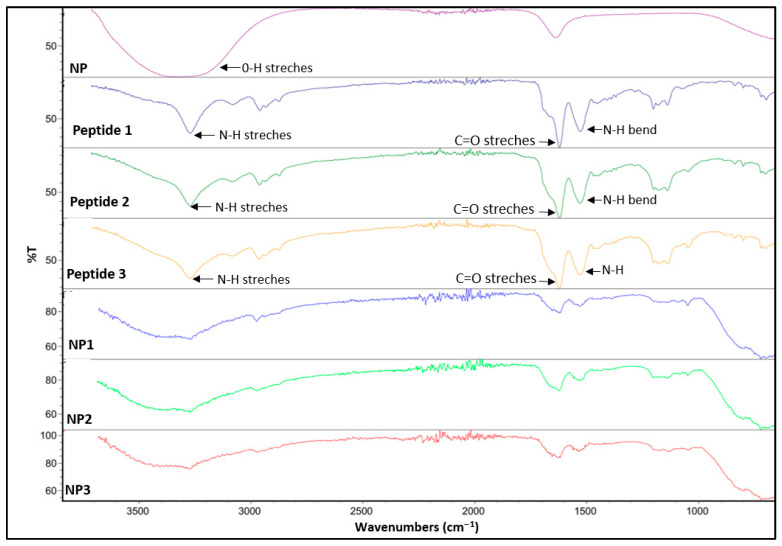
FT-IR spectra of bare aluminum oxide nanoparticle (**NP**), free peptides (**1**–**3**), and peptide conjugated aluminum oxide nanoparticles (**NP1**–**NP3**).

Moreover, Fourier-transform infrared (FTIR) spectroscopy was conducted to further confirm the successful synthesis of the peptides and surface modification of nanoparticles with peptides. FT-IR spectra of free peptides **1**–**3** showed characteristic absorption bands for amide bond formation. Notably, strong bands corresponding to the amide (C=O stretching) and (N–H bending) vibrations were observed around 1650 cm^−1^ and 1540 cm^−1^, respectively, consistent with peptide backbone formation. Additional peak at 3300 cm^−1^ confirmed the presence of N–H stretching vibrations. Significant changes observed in the FT-IR spectra of the peptide conjugated aluminum oxide nanoparticles (**NP1**–**NP3**), compared to the bare aluminum oxide nanoparticles (**NP**), indicate successful surface functionalization. Specifically, the disappearance of the broad O–H stretching band around 3400 cm^−1^ and the appearance of new amide bands at approximately 1650 cm^−1^ and 1540 cm^−1^, respectively, confirm the effective attachment of the newly synthesized peptides onto the aluminum oxide nanoparticle surface (Figure 5). These spectral changes indicate the formation of hydrogen bonds and electrostatic interactions between the carboxyl, amine, and amide groups of the peptides and the hydroxylated Al_2_O_3_ surface.

Next, zeta potential analysis was employed to evaluate the surface charge of both unmodified and peptide-functionalized nanoparticles. The zeta potentials of unmodified (**NP**) and modified aluminum oxide nanoparticles (**NP1**–**3**) were measured as –2.2 ± 0.7, 12.0 ± 0.93, 14.6 ± 1.14, and 12.8 ± 0.99 mV, respectively (Figure S6). These results demonstrate a significant shift from a negative surface charge in the unmodified nanoparticles (**NP**) to positive values following peptide conjugation. This notable increase in zeta potential confirms the successful surface modification of the nanoparticles with peptides **1**–**3**, reflecting both the presence of positively charged peptide moieties and an overall enhancement in surface charge density. This change in surface charge may affect how the nanoparticles interact with biological systems.

### 3.3. Biological Studies

#### 3.3.1. Antimicrobial Activity

The antimicrobial activities of our compounds, namely the bare aluminum nanoparticle (**NP**), free peptides (**1**–**3**), and peptide-conjugated nanoparticles (**NP1**–**3**), were evaluated against selected model microorganisms, and the minimum inhibitory concentration (MIC) values are summarized in Table 2. Our results indicate that the peptide-conjugated nanoparticles (**NP1**–**NP3**) exhibit enhanced antimicrobial activity compared to both the free peptides and the bare aluminum nanoparticle. In general, **NP1**–**NP3** were more effective against bacterial strains than against fungal strains.

Among the tested nanoconjugates, **NP1** demonstrated the strongest antimicrobial effect, with an MIC of 16 mg/L against *E. coli*, *P. aeruginosa*, and *L. pneumophila* subsp. *pneumophila*. It also exhibited moderate activity against *S. aureus* and *B. spizizenii*, with MIC values of 32 mg/L. **NP2** showed its lowest MIC value of 16 mg/L against *B. spizizenii*, *E. faecalis*, and *L. pneumophila*, while **NP3** achieved the same MIC (16 mg/L) against *B. spizizenii* and *E. faecalis*.

In contrast, the free peptides (**1**–**3**) and the bare nanoparticle (**NP**) exhibited com-paratively weaker antimicrobial effects, with MIC values ranging from 32 to 256 mg/L across all tested strains. Ampicillin and fluconazole were used as control antibiotics, and all tested compounds displayed lower antimicrobial activity than these controls.

These results suggest that the positive charges of peptides and peptide-conjugated nanoparticles allow them to interact with the negatively charged bacterial membrane, leading to membrane disruption and thus bacterial growth inhibition or death. The lower MIC values observed for the peptide–aluminum conjugates support our prediction that the uniform conjugation of two active species into a single entity can yield enhanced antimicrobial activity. This finding suggests that **NP1**–**NP3** may promote the bacterial cell membranes to develop larger or perhaps more pores, resulting in internalization of the peptide-alumina nanostructures into the bacteria. By stimulating the production and release of a regionally high concentration of reactive oxygen species (ROS), the internalized peptide-alumina nanoconjugate may further damage bacterial subcellular components, ultimately causing bacterial cell death [28]. This enhanced antimicrobial action may also be attributed to the specific physicochemical interactions between the peptides and the alumina nanoparticles. Electrostatic attraction and hydrogen bonding may stabilize the peptides on the nanoparticle surface, preventing their degradation. In future studies, the inclusion of mechanistic controls such as ROS scavengers or metal chelators may be necessary to help clarify the antimicrobial mechanisms of these nanoconjugates. Furthermore, the increased local concentration of peptides on the nanoparticle surface may facilitate more effective interactions with bacterial membranes, enhancing membrane disruption and cellular uptake.

Our results align with prior work on peptide-functionalized nanoparticles for antimicrobial applications, such as bioactive compound-peptide conjugates [29], phage-mimicking particles [30], and peptide-conjugated chitosan [31]. However, our work uniquely employs aluminum oxide nanoparticles as a robust inorganic platform for peptide immobilization, offering a distinct surface chemistry and potential enhanced antimicrobial effects. While these studies have reported antimicrobial systems based on organic or polymeric nanoparticles functionalized with peptides or bioactive molecules, our work introduces a distinct inorganic–organic hybrid approach. Our system takes advantage of the unique surface chemistry and stability of Al_2_O_3_ to attach free antimicrobial peptides. In parallel, the preparation of nickel, silver, and copper metal oxide nanostructures containing proteins and peptides has also been previously reported, indicating the growing interest in biologically derived hybrid materials [32]. However, in our study, we use aluminum oxide (Al_2_O_3_) nanoparticles as an inorganic support material for peptide immobilization.

#### 3.3.2. Biofilm Inhibition Activity

**Figure 6:**
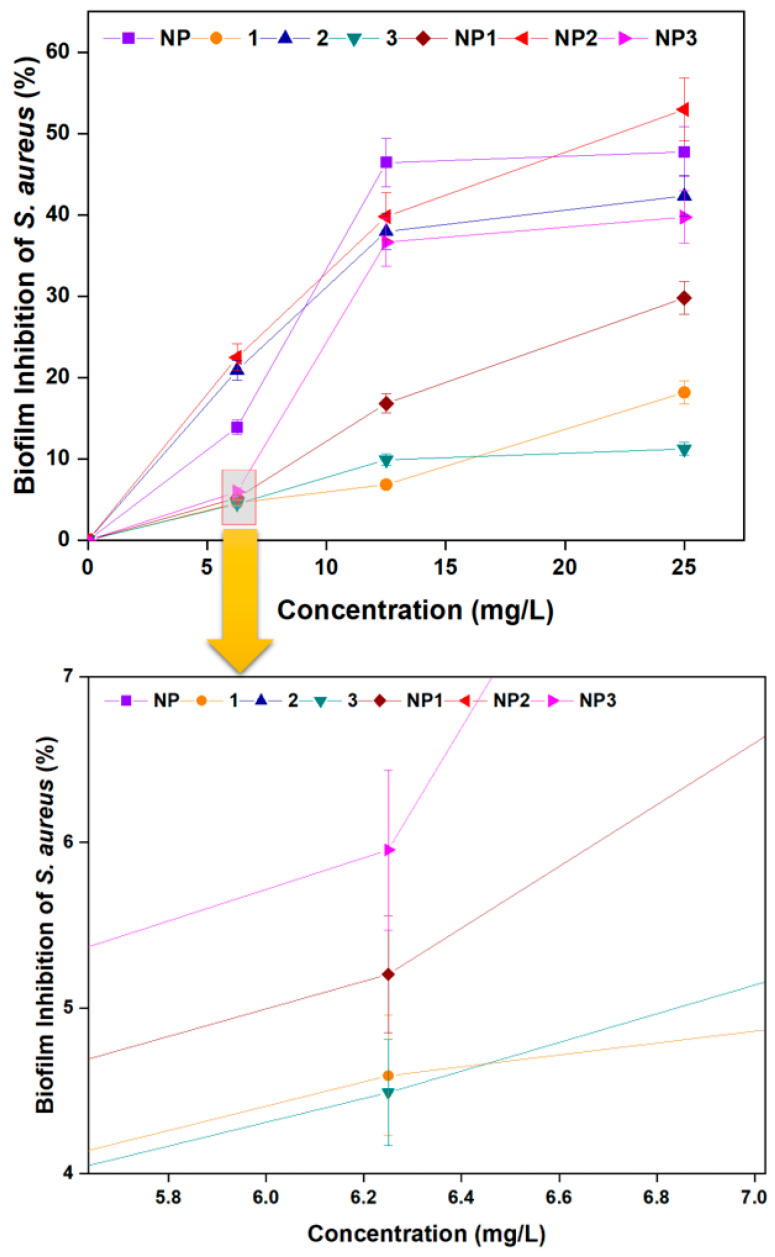
Biofilm inhibition of *S. aureus* by free peptides (**1**–**3**), bare aluminum nanoparticle (**NP**), and peptide–aluminum conjugates (**NP1**–**NP3**).

**Figure 7:**
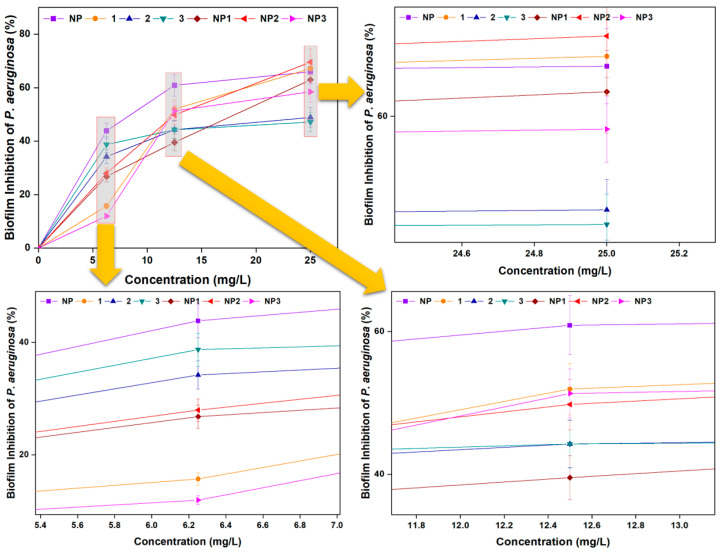
Biofilm Inhibition of *P. aeruginosa* by free peptides (**1**–**3**), bare aluminum nanoparticle (**NP**), and peptide–aluminum conjugates (**NP1**–**NP3**).

The capacity of *S. aureus* and *P. aeruginosa* to generate biofilms, which are complex microbial populations encased in an extracellular matrix that sticks to surfaces, is well-known. Due to their strong resilience to host immune responses and antimicrobial therapies, bacterial biofilms present serious therapeutic problems [33]. Biofilm generation is a major factor in the persistence of chronic infections and is often linked to infections linked to medical devices [34]. Finding substances that can prevent the formation of biofilms is becoming more and more important due to the challenges in treating infections linked to biofilms. Therefore, in this study, the capacity of **NP**, **1**–**3**, and **NP1**–**NP3** to inhibit biofilm formation by *S. aureus* and *P. aeruginosa* was assessed. Data on the inhibition of *S. aureus* and *P. aeruginosa* biofilms are presented in Figure 6 and Figure 7, respectively.

As the concentration increased from 6.25 mg/L to 25 mg/L, the inhibition of *S. aureus* biofilm formation also increased (Figure 6). At 25 mg/L, the inhibition percentages were as follows: **NP** increased from 13.88% to 47.76%; peptide **1** from 4.59% to 18.16%; peptide **2** from 20.98% to 42.35%; peptide **3** from 4.49% to 11.22%; **NP1** from 5.20% to 29.80%; **NP2** from 22.48% to 52.98%; and **NP3** from 5.95% to 39.73%.

A similar trend was observed for *P. aeruginosa* biofilm inhibition, which also increased with rising concentration (Figure 7). At 25 mg/L, the inhibition percentages were as follows: **NP** increased from 43.84% to 65.96%; peptide **1** from 15.71% to 67.15%; peptide **2** from 34.19% to 48.87%; peptide **3** from 38.71% to 47.13%; **NP1** from 26.80% to 62.92%; **NP2** from 27.96% to 69.55%; and **NP3** from 11.94% to 58.47%.

Overall, these results show that all compounds exhibited concentration-dependent biofilm inhibition activity. Notably, **NP2** was the most effective in inhibiting biofilm formation for both bacterial strains, highlighting the potential synergistic effect of peptide–nanoparticle conjugation in biofilm control.

Similar antibiofilm effects have been reported for peptide-loaded chitosan nanoparticles [35], chitosan-based pexiganan conjugates [36], and gold nanoparticle–peptide conjugates [37]. Unlike these organic or gold-based systems, our study uses an inorganic Al_2_O_3_ core. An antimicrobial mechanism was reported by Alhosani et al., where ZnO–Ag nanoparticles reduced *P. aeruginosa* biofilms by damaging bacterial membranes and producing reactive oxygen species (ROS), particularly superoxide and methyl radicals [38]. In this context, our alumina–peptide nanoparticles may work in a similar way, combining physical damage and oxidative stress to disrupt biofilms.

#### 3.3.3. Microbial Cell Viability Inhibition

**Figure 8:**
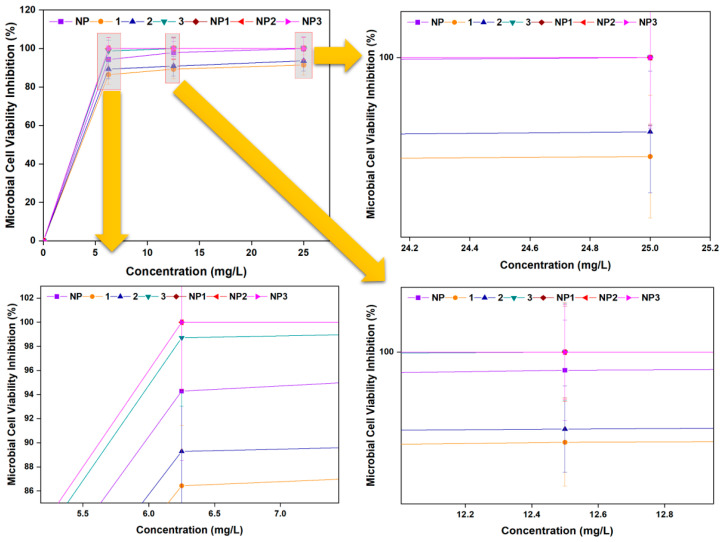
*E. coli* viability inhibition activity by free peptides (**1**–**3**), bare aluminum nanoparticle (**NP**), and peptide–aluminum conjugates (**NP1**–**3**).

The *E. coli* cell viability inhibition capacities of the bare nanoparticle (**NP**), free peptides (1 to 3), and peptide-conjugated nanoparticles (**NP1** to **NP3**) were evaluated, and the results are presented in Figure 8. At 6.25 mg/L, inhibition levels were 94.28% for **NP**, 86.43% for peptide 1, 89.29% for peptide 2, 98.71% for peptide 3, and 100% for **NP1**, **NP2**, and **NP3**. At 25 mg/L, peptides 1 and 2 showed inhibition levels exceeding 90%, while all other samples achieved complete inhibition (100%) of *E. coli* viability. These results indicate that while all compounds demonstrated strong antimicrobial effects, the peptide-conjugated nanoparticles were the most effective in reducing bacterial viability.

These findings agree with other recent studies showing that different peptide-functionalized nanomaterials, such as chitosan-based nanoparticles [36], gold nanoparticle conjugates [37], and peptide-loaded nanocellulose [39], can effectively inhibit bacterial growth. Our results add to this evidence by demonstrating strong antimicrobial activity using aluminum oxide nanoparticles combined with peptides. This suggests that peptide-conjugated nanoparticles, across various material types, could be promising alternatives to traditional antibiotics. Further research, including in vitro and in vivo toxicologic assays, is needed to fully explore their potential.

#### 3.3.4. DNA Cleavage Activity

**Figure 9:**
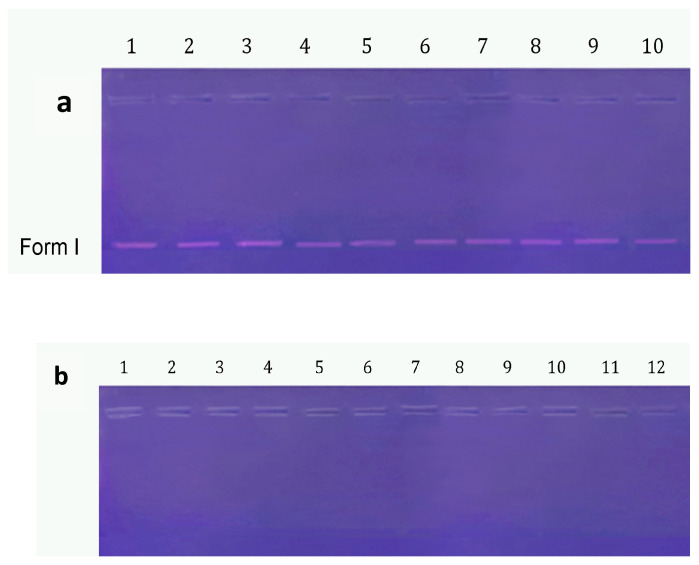
(**a**) The DNA cleavage activity of free peptides 1-3; Lane (1): DMSO + pBR 322 DNA; Lane (2): 32 mg/L of **1** + pBR 322 DNA; Lane (3): 64 mg/L of **1** + pBR 322 DNA; Lane (4): 128 mg/L of **1** + pBR 322 DNA; Lane (5): 32 mg/L of **2** + pBR 322 DNA; Lane (6): 64 mg/L of **2** + pBR 322 DNA; Lane (7): 128 mg/L of **2** + pBR 322 DNA; Lane (8): 32 mg/L of **3** + pBR 322 DNA; Lane (9): 64 mg/L of **3** + pBR 322 DNA; Lane (10): 128 mg/L of **3** + pBR 322 DNA. (**b**) The DNA cleavage activity of bare aluminum nanoparticle NP, and peptide-modified aluminum nanoparticles, NP 1–3; Lane (1): 32 mg/L of NP + pBR 322 DNA; Lane (2): 64 mg/L of NP + pBR 322 DNA; Lane (3): 128 mg/L of NP + pBR 322 DNA; Lane (4): 32 mg/L of NP1 + pBR 322 DNA; Lane (5): 64 mg/L of NP1 + pBR 322 DNA; Lane (6): 128 mg/L of NP1 + pBR 322 DNA; Lane (7): 32 mg/L of NP2 + pBR 322 DNA; Lane (8): 64 mg/L of NP2 + pBR 322 DNA; Lane (9): 128 mg/L of NP2 + pBR 322 DNA; Lane (10): 32mg/L of NP3+ pBR 322 DNA; Lane (11): 64 mg/L of NP3 + pBR 322 DNA; Lane (12): 128 mg/L of NP3 + pBR 322 DNA.

In addition to antimicrobial, and antibiofilm assays, DNA cleavage activity was investigated to explore whether the tested compounds could induce genetic damage, which may contribute to their antimicrobial mechanism or suggest broader biological applications. Therefore, our compounds **NP**, **1**–**3**, and **NP1**–**NP3** were investigated for their DNA cleavage effects (Figure 9). DNA was tracked through gel electrophoresis as it changes from its supercoiled form I (SC) to its linear form III (LC) and nicked circular form II (NC). As shown in Figure 9a, the free peptides (1–3) did not induce any observable DNA cleavage, as the supercoiled (SC) form remained intact. In contrast, the bare nanoparticle (**NP**) and all peptide-conjugated nanoparticles (**NP1**–**NP3**) caused complete fragmentation of the DNA. The absence of any visible bands in these samples suggests that the DNA was not merely converted into nicked circular (NC) or linear (LC) forms but was likely broken down into very small oligonucleotides that could not be detected by gel electrophoresis.

Our results align with previous findings where an oligocationic Cu(II) peptide helix selectively bound and cleaved DNA junctions via a ROS-mediated oxidative damage pathway [40]. Similarly, bioactive α-hydroxy carboxylate-modified MnFe_2_O_4_ nanoparticles were reported to induce DNA cleavage, converting plasmid DNA from supercoiled to linear and nicked forms with good efficiency [41]. Unlike these studies, which observed partial DNA cleavage forms, our nanoparticles caused extensive DNA breakdown into small fragments undetectable by gel electrophoresis.

Overall, the strong DNA cleavage activity observed for **NP** and **NP1**–**NP3** highlights their potential not only as antimicrobial agents but also as candidates for future exploration in anticancer applications, following further toxicologic studies and in vivo tests.

#### 3.3.5. Antioxidant Activity

While the main goal of this study was to investigate the antimicrobial and antibiofilm effects of the synthesized compounds, their antioxidant potential was also evaluated to explore possible additional biological benefits. Antioxidants are important in protecting cells from damage caused by reactive oxygen species (ROS) and reactive nitrogen species (RNS), which are unstable molecules naturally produced in the body. If not neutralized, these free radicals can damage proteins, lipids, and DNA, contributing to infections, inflammation, and various diseases [42,43]. Compounds with both antimicrobial and antioxidant properties may provide a more balanced therapeutic effect.

**Figure 10:**
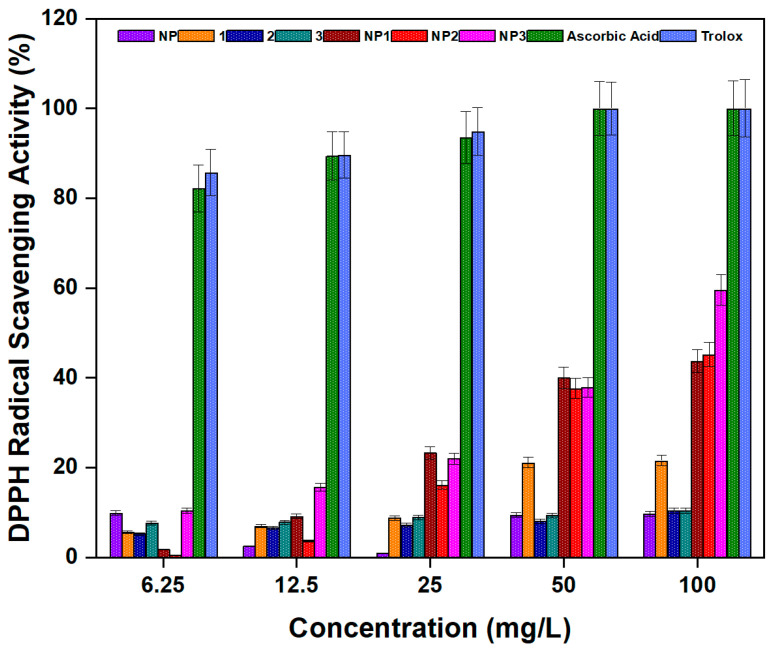
Antioxidant activity of free peptides (**1**–**3**), bare aluminum nanoparticle (**NP**), and peptide–aluminum conjugates (**NP1**–**NP3**).

With this in mind, the antioxidant activities of **NP**, peptides **1**–**3**, and **NP1**–**3** were tested (Figure 10). Antioxidant activity increased with concentration, and the peptide-conjugated nanoparticles (**NP1**–**NP3**) showed significantly higher activity than the bare nanoparticle or free peptides. At a concentration of 12.5 mg/L, the antioxidant activities of **NP**, peptides **1**–**3**, **NP1**–**NP3**, ascorbic acid, and Trolox were determined to be 2.47%, 6.97%, 6.52%, 7.83%, 9.13%, 3.72%, 15.65%, 89.43%, and 89.62%, respectively. When the concentration was increased to 100 mg/L, the corresponding antioxidant activities of our samples and the standard compounds rose to 9.66%, 21.57%, 10.43%, 10.50%, 43.70%, 45.22%, 59.57%, 100%, and 100%, respectively.

These results suggest that **NP1**–**NP3** possess not only strong antimicrobial properties but also notable antioxidant capacity. This dual activity may enhance their potential as multifunctional agents for biomedical or environmental applications. The good antioxidant capacity of **NP1**–**NP3** compounds can be attributed to their ability to donate electrons or hydrogen atoms to stabilize and quench reactive and harmful free radicals. In addition, conjugating the peptides to alumina nanoparticles may help maintain the active structure of the peptides and prevent their degradation, allowing them to function more effectively. The large surface area of the nanoparticles may also support better exposure of antioxidant groups, enhancing their free radical scavenging ability.

#### 3.3.6. Amylolytic Activity

Although the primary focus of this study was on antimicrobial and antioxidant properties, the amylolytic activity of the compounds was also investigated to explore their broader biotechnological potential. Enzymes such as α-amylase play key roles in various industrial processes by breaking down starch into smaller sugar units [44]. Enhancing amylase activity through nanomaterials has gained attention in fields ranging from food processing to biomedical applications [45].

**Figure 11:**
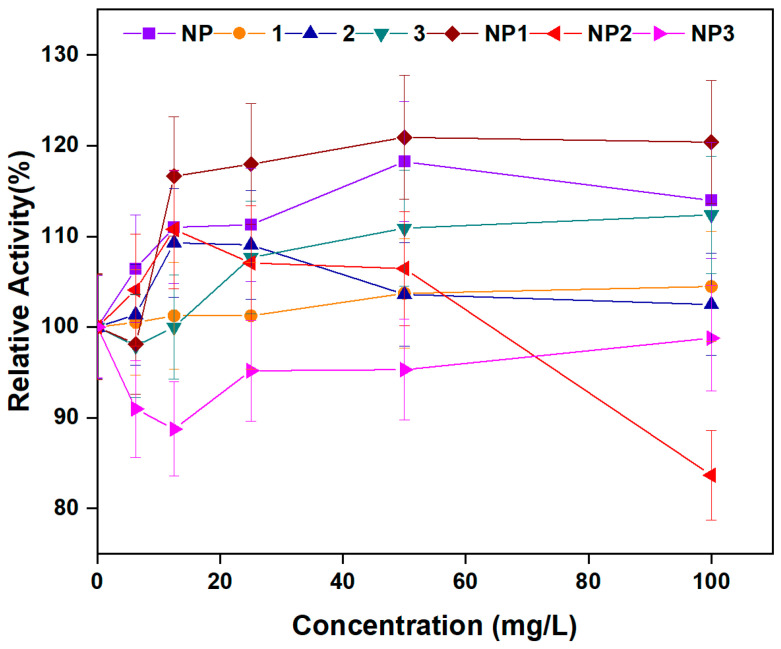
Amylolytic activity of free peptides (**1**–**3**), bare aluminum nanoparticle (**NP**), and peptide–aluminum conjugates (**NP1**–**NP3**).

In this context, the effects of **NP**, peptides **1**–**3**, and **NP1**–**3** on α-amylase activity were evaluated (Figure 11). While compounds **NP**, peptide **1**–**3**, **NP1**, and **NP2** showed an enhancing effect on α-amylase activity, **NP3** did not contribute to the activity. At a concentration of 12.5 mg/L, α-amylase activity was increased by 10.99%, 1.24%, 9.28%, 1.0%, 16.62%, and 10.77% with **NP**, peptides **1–3** and **NP1**, and **NP2**, respectively. When the concentration was raised to 50 mg/L, α-amylase activities were increased by 13.94%, 4.46%, 2.48%, 12.38%, 20.38%, and 6.44% by **NP**, **1**–**3** and **NP1**, and **NP2**, respectively. Overall, the observed increase in enzyme activity was generally concentration-dependent.

Taken together, results of our study suggest that **NP**, peptides **1**–**3**, **NP1**, and **NP2** may find applications in the enzyme industry due to their ability to enhance amylolytic activity. Although unrelated to their antimicrobial function, the amylolytic activity of these compounds highlights their potential as multifunctional agents with relevance in both biomedical and industrial enzyme applications.

Supporting our findings, previous studies have shown that conjugating α-amylase with metal nanoparticles can significantly enhance its activity. For example, bioconjugation with silver nanoparticles increased reducing sugar production by 2.5 times compared to free α-amylase [46]. Similarly, α-amylase activity in starch stain removal improved by 60% with silver nanoparticles and 37% with gold nanoparticles [47].

## 4. Conclusions

In summary, this study reports the development of a novel nanoformulation based on antimicrobial peptides conjugated to aluminum nanoparticles. Peptide conjugation significantly enhanced the biological performance of the nanoparticles, resulting in improved antimicrobial potency, as evidenced by lower MIC values compared to the free peptides and bare aluminum. The conjugated nanoparticles also exhibited strong antibiofilm activity, effectively inhibiting biofilm formation by *S. aureus* and *P. aeruginosa*. Taken together with these findings, our results in this study suggest that peptide-conjugated nanoparticles may be an effective alternative to traditional antibiotic treatments and should be investigated in detail following in vitro and in vivo toxicologic assays.

Cell viability assays further confirmed their strong bactericidal effect, showing complete inhibition of *E. coli* growth at tested concentrations. Overall, the enhanced biological activities of these peptide–aluminum nanoformulations position them as promising candidates for treating biofilm-associated infections. 

Beyond their antimicrobial properties, the peptide-conjugated nanoparticles (**NP1**–**NP3**) demonstrated additional biofunctionalities, including increased antioxidant capacity, α-amylase activation, and DNA cleavage activity, suggesting a multifunctional platform as promising candidates for further biological evaluation.

To further evaluate their potential applicability, the long-term stability of the peptide-conjugated nanoparticles should be assessed under various environmental conditions, including fluctuations in pH, temperature, and extended storage durations. Future studies should also focus on evaluating in vivo efficacy and conducting comprehensive safety profiling. These investigations will be essential for advancing these materials toward clinical or environmental applications.

## Figures and Tables

**Table 1:** Calculated and observed *m*/*z* values for the ion fragments of peptides **1**–**3.**

	Peptide 1		Peptide 2		Peptide 3	
	Calculated Mass (Da)	Observed Mass (Da)	Calculated Mass (Da)	Observed Mass (Da)	Calculated Mass (Da)	Observed Mass (Da)
M	1394.980	-	1278.670	-	1280.900	-
[M + 1H]^+^	1395.987	-	1279.677	-	1281.907	-
[M + 2H]^2+^	698.497	698.031	640.342	640.469	641.457	641.960
[M + 3H]^3+^	466.001	466.034	427.231	427.281	427.974	427.974
[M + 4H]^4+^	349.752	-	320.675	-	321.232	-

**Table 2:** Antimicrobial Activity of free peptides (**1**–**3**), bare aluminum nanoparticle (**NP**), and peptide–aluminum conjugates (**NP1**–**3**).

	MIC Values (mg/L)								
Microorganisms	1	2	3	NP	NP1	NP2	NP3	AMP *	FLZ *
*Staphylococcus aureus*	64	64	128	128	32	64	32	0.5	-
*Bacillus spizizenii*	64	32	64	128	32	16	16	0.5	-
*Enterococcus faecalis*	64	32	128	128	64	16	16	0.5	-
*Escherichia coli*	32	32	64	64	16	32	32	0.5	-
*Pseudomonas aeruginosa*	32	32	64	64	16	32	32	0.5	-
*Legionella pneumophila*	64	32	32	128	16	16	32	0.5	-
*Candida albicans*	128	128	128	256	128	64	64	-	1
*Candida glabrata*	128	64	256	256	64	64	64	-	1

* (AMP: Ampicillin; FLZ: Fluconazole).

## Data Availability

The raw data supporting the conclusions of this article will be made available by the authors on request.

## Supplementary Materials

The supporting information can be downloaded at: https://www.mdpi.com/article/10.3390/pharmaceutics17111490/s1, Figure S1: HPLC chromatogram and MS/MS spectrum of crude peptide 1; Figure S2: HPLC chromatogram and MS/MS spectrum of crude peptide 2; Figure S3: HPLC chromatogram and MS/MS spectrum of crude peptide 3; Figure S4: The FESEM image (**a**) and elemental mapping analysis (**b**) of unmodified aluminum oxide nanoparticles (**NP**); Figure S5: The FESEM image (**a**) and elemental mapping analysis (**b**) of modified aluminum oxide nanoparticles (**NP1**); Figure S6: Zeta potential distribution of **NP** (green); **NP1** (yellow); **NP2** (red); **NP3** (blue).
