# Supplementary material for: Design of Peptide-Modified Aluminum Nanoparticles with Enhanced Antimicrobial, Antibiofilm, Antioxidant, and DNA-Cleaving Properties
Source: Pharmaceutics. 2025 Nov 19;17(11):1490. doi: 10.3390/pharmaceutics17111490 (PMC12655476; doi:10.3390/pharmaceutics17111490)
Supplement: Supplementary file 1 [file pharmaceutics-17-01490-s001.zip › pharmaceutics-3963924-supplementary.pdf]

Supplementary Materials: Design of Peptide–Modified Alumina Nanoparticles with Enhanced Antimicrobial, Antibiofilm, Antioxidant, and DNA-Cleaving Properties

Zeynep Kanlidere[1,*], Nazlı Farajzadeh Öztürk[2], M. Serkan Yalçın[3], Sadin Özdemir[4]

1 *Department of Pharmaceutical Basic Sciences, Faculty of Pharmacy, Acibadem Mehmet Ali Aydinlar University, Istanbul, Türkiye;* zeynep.kanlidere@acibadem.edu.tr
2 *Department of Analytical Chemistry, Faculty of Pharmacy, Acibadem Mehmet Ali Aydinlar University, Istanbul, Türkiye;* nazli.ozturk@acibadem.edu.tr
3 *Department of Chemistry and Chemical Processing Technologies, Technical Science Vocational School, Mersin University, 33343, Mersin, Türkiye* serkanyalcin@mersin.edu.tr
4 *Food Processing Programme, Technical Science Vocational School, Mersin University, TR-33343 Yenisehir, Mersin, Türkiye;* sadinozdemir@mersin.edu.tr

* Correspondence zeynep.kanlidere@acibadem.edu.tr

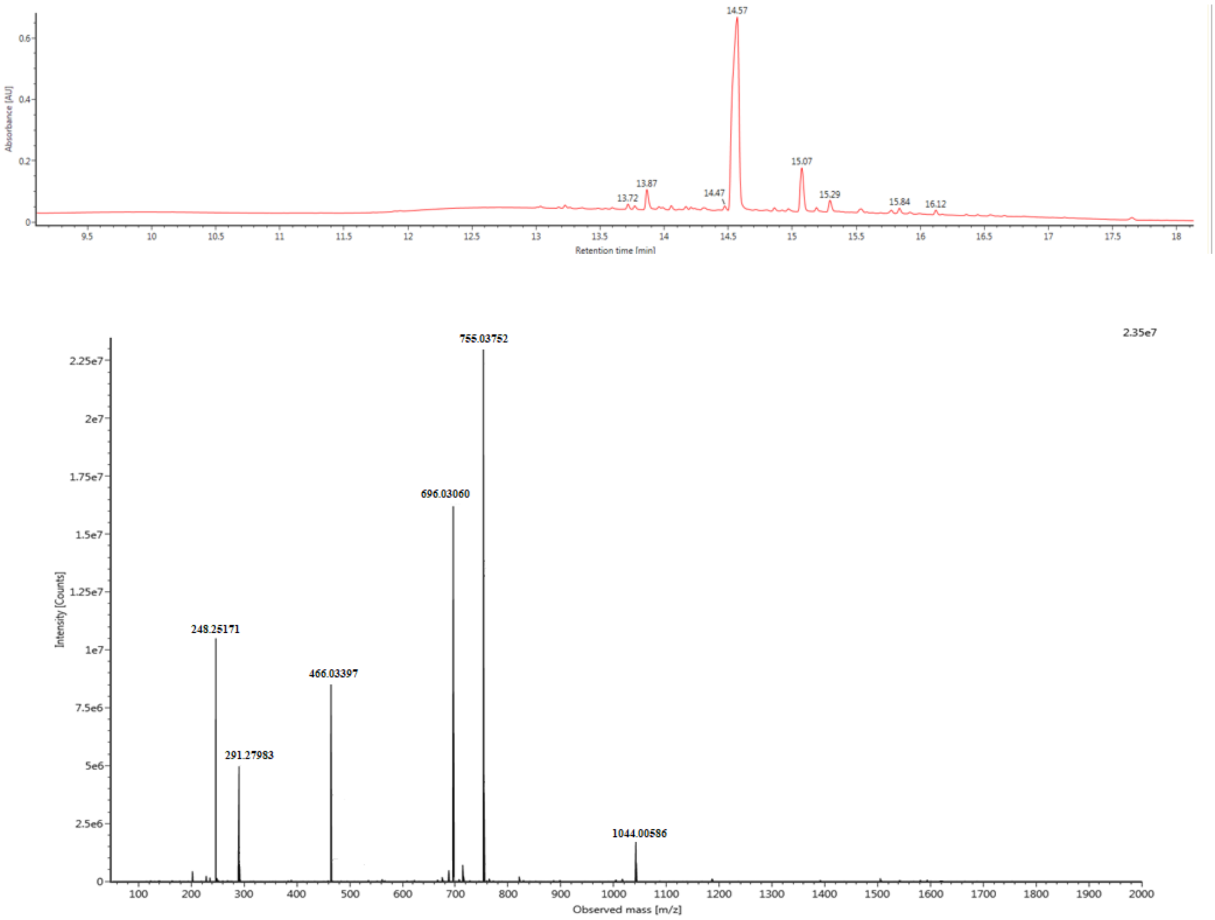

Figure S1. HPLC chromatogram and MS/MS spectrum of crude peptide 1; calculated: 698.497, found: 698.031 $[M+2H]^{2+}$; calculated: 466.001, found: 466.034 $[M+3H]^{3+}$.

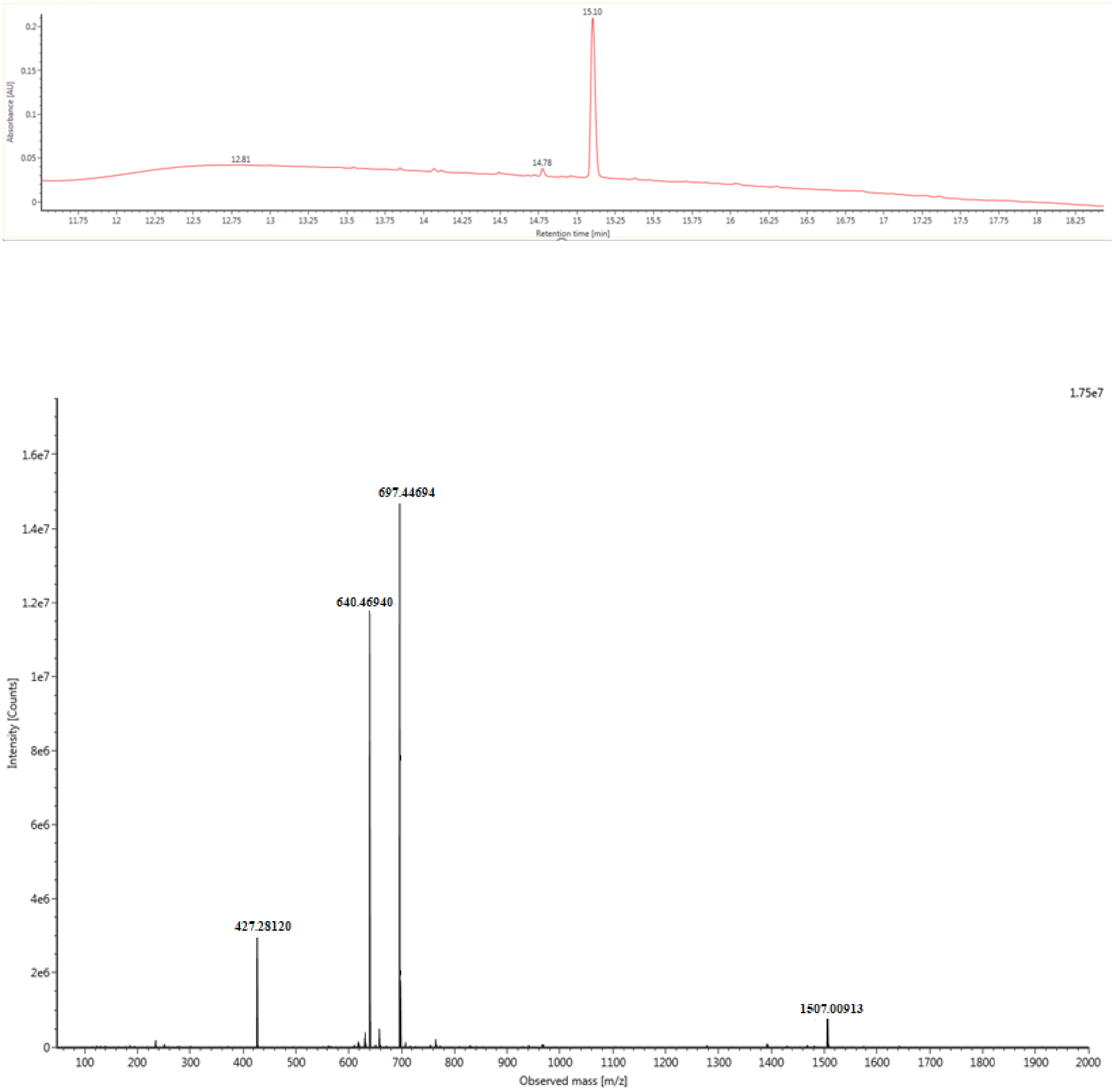

Figure S2. HPLC chromatogram and MS/MS spectrum of crude peptide 2; calculated: 640.342, found: 640.469 $[M+2H]^{2+}$; calculated: 427.031, found: 427.231 $[M+3H]^{3+}$.

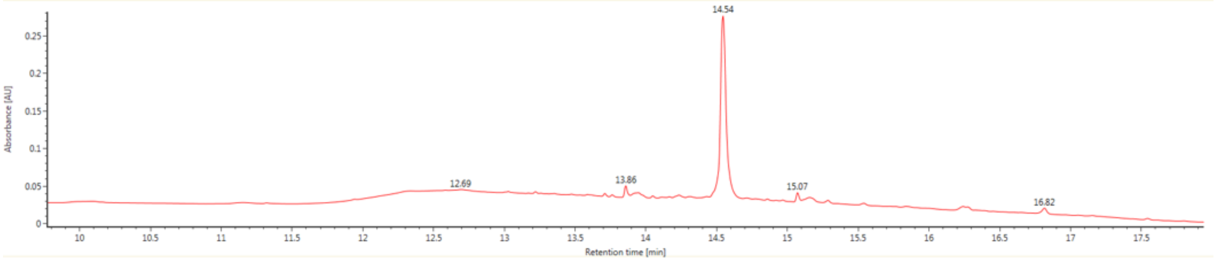

,

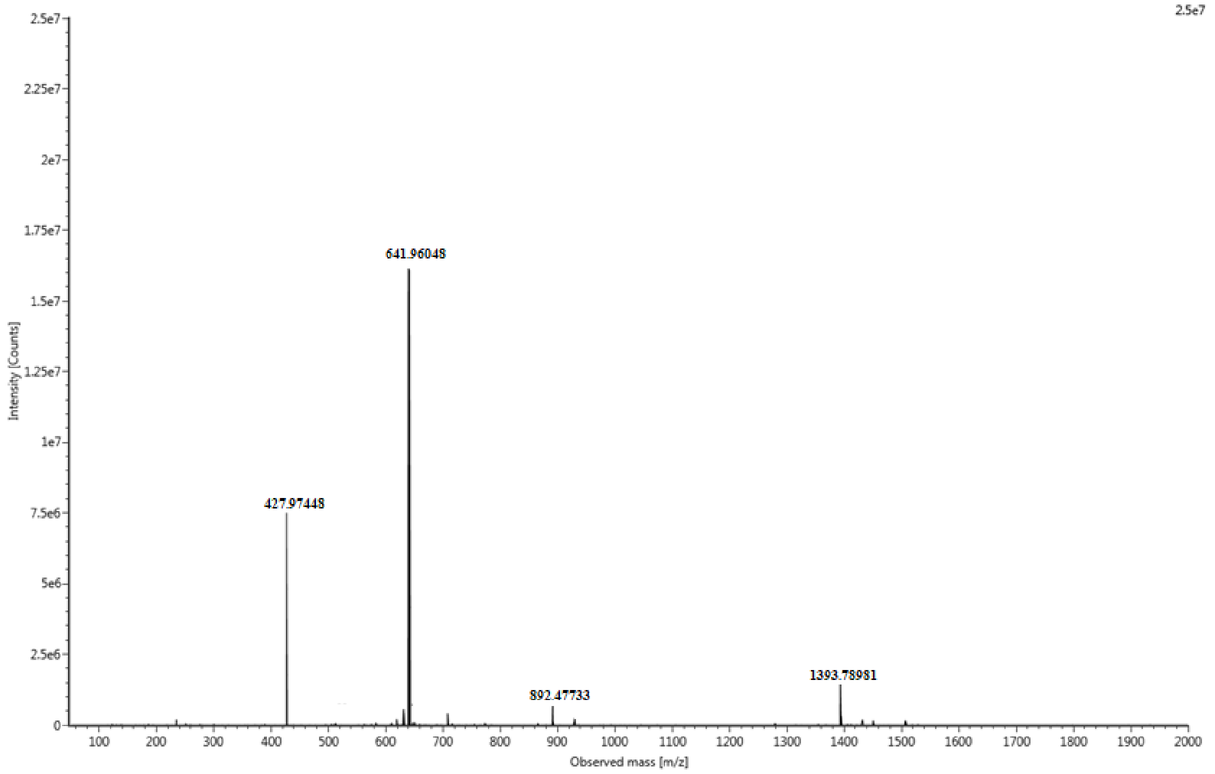

Figure S3. HPLC chromatogram and MS/MS spectrum of crude peptide 3; calculated: 641.457, found: 641.960 $[M+2H]^{2+}$; calculated: 427.974, found: 427.974 $[M+3H]^{3+}$.

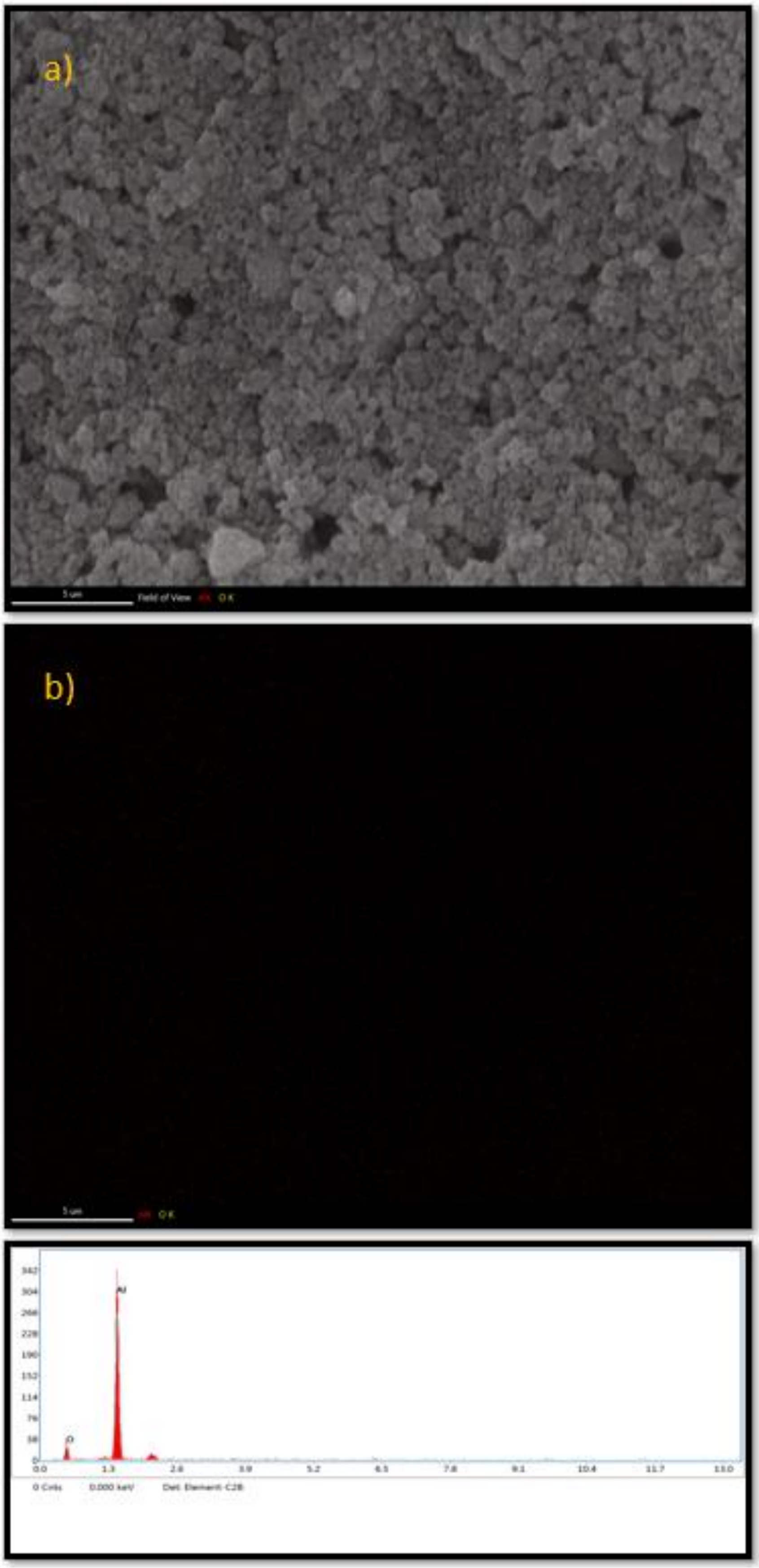

Figure S4. The FESEM image (a) and elemental mapping analysis (b) of unmodified aluminum oxide nanoparticles (**NP**).

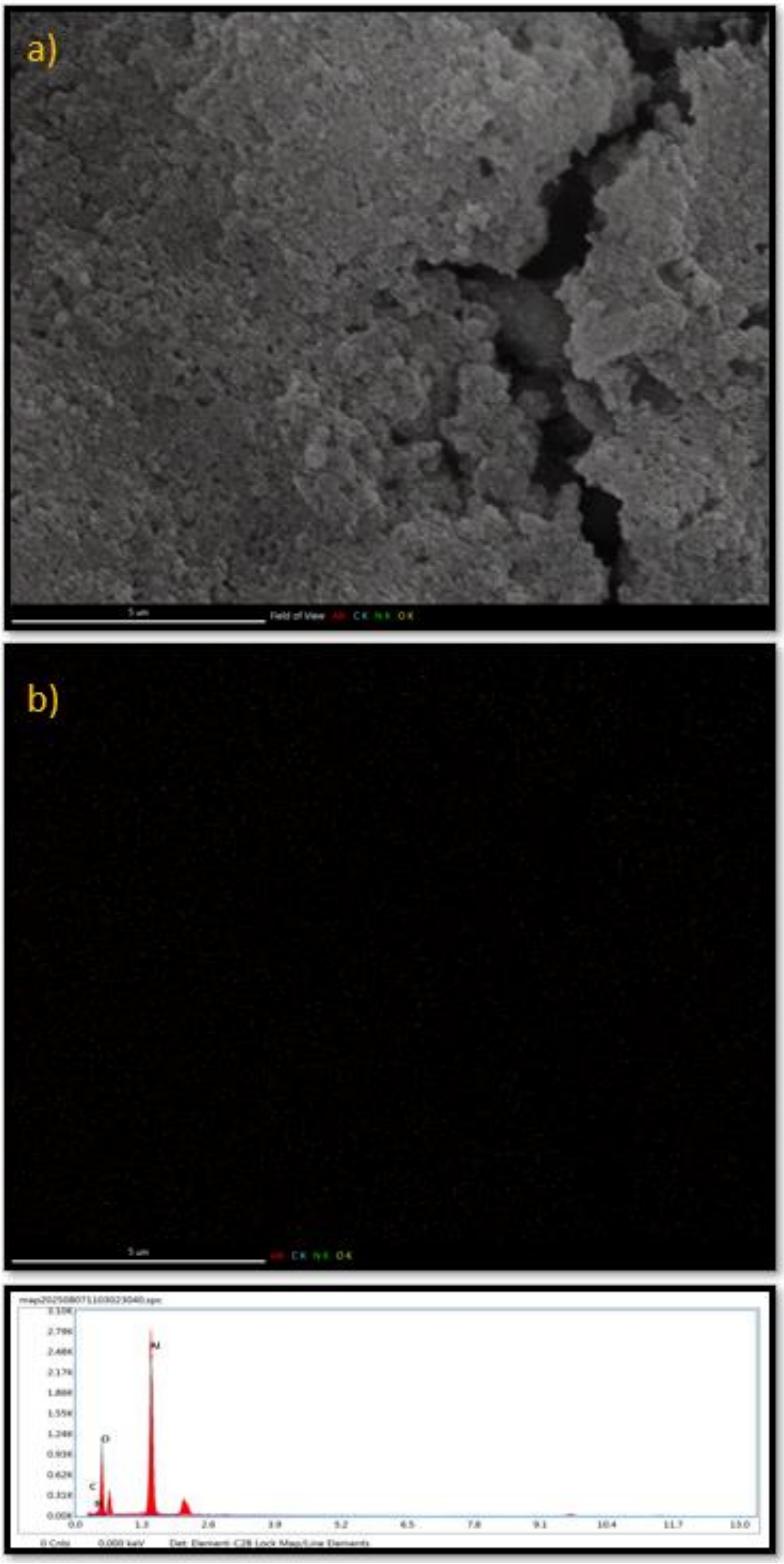

Figure S5. The FESEM image (a) and elemental mapping analysis (b) of modified aluminum oxide nanoparticles (**NP1**).

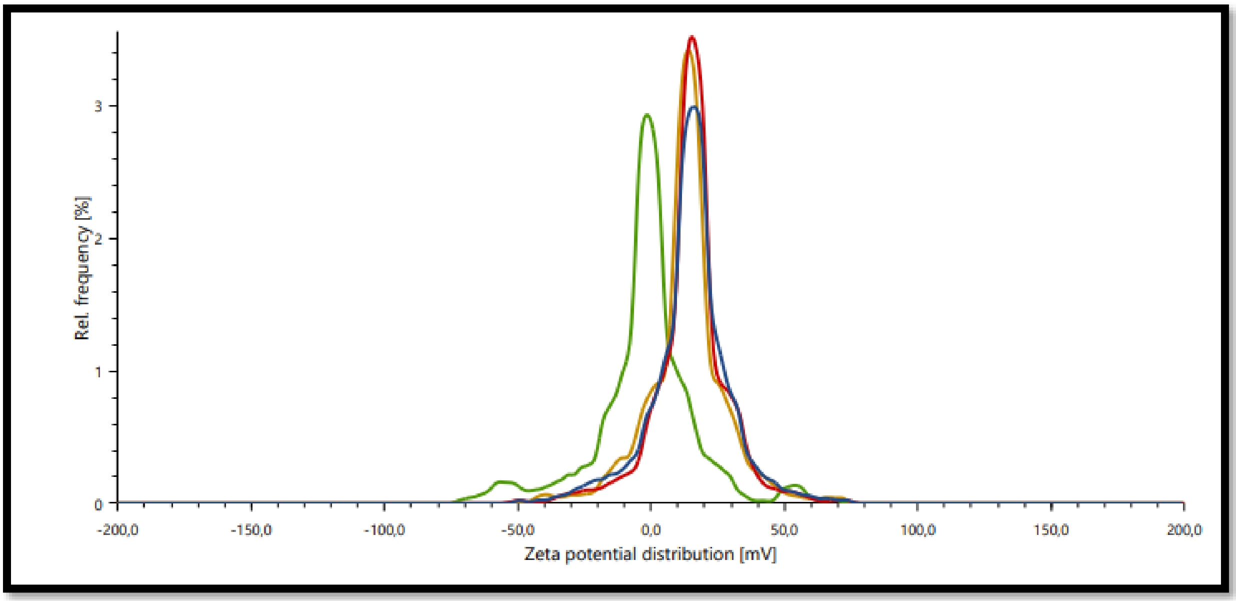

Figure S6. Zeta potential distribution of **NP** (green); **NP1** (yellow); **NP2** (red); **NP3** (blue).
